# Parenting styles, maladaptive coping styles, and disturbed eating attitudes and behaviors: a multiple mediation analysis in patients with feeding and eating disorders

**DOI:** 10.7717/peerj.14880

**Published:** 2023-02-10

**Authors:** Mohsen Khosravi, Seyed Teymur Seyedi Asl, Alireza Nazari Anamag, Milad SabzehAra Langaroudi, Jafar Moharami, Sadegh Ahmadi, Alireza Ganjali, Zahra Ghiasi, Mohammad Nafeli, Rashya Kasaeiyan

**Affiliations:** 1 Department of Psychiatry and Clinical Psychology, Zahedan University of Medical Sciences, Zahedan, Iran; 2 Department of Psychology, Mohaghegh Ardabili University, Ardabil, Iran; 3 Department of Humanities and Social Sciences, Science and Research Branch of Islamic Azad University, Tehran, Iran; 4 Department of Psychology, Rahman Institute of Higher Education, Ramsar, Iran; 5 Department of Psychology, Islamic Azad University, North Tehran, Tehran, Iran; 6 Department of Education, Farhangian University, Zanjan, Iran; 7 Health Promotion Research Center, Zahedan University of Medical Sciences, Zahedan, Iran; 8 Zahedan University of Medical Sciences, Zahedan, Iran; 9 Department of Clinical Psychology, Shiraz University of Medical Sciences, Shiraz, Iran

**Keywords:** Eating attitudes and behaviors, Feeding and eating disorders, Maladaptive coping styles, Parenting styles

## Abstract

**Background:**

Although preliminary studies support the roles of unhealthy parenting styles and maladaptive coping styles in increasing rates of disturbed eating attitudes and behaviors (EAB) and clinically significant feeding and eating disorders (FED), underlying mechanisms have not been well-recognized. This study aims to investigate the factors associated with disturbed EAB and the mediating roles of overcompensation and avoidance coping styles in the relationship between different types of parenting styles and disturbed EAB among patients with FED.

**Methods:**

A total of 102 patients with FED in Zahedan, Iran, participating in this cross-sectional study (from April to March 2022) completed a sociodemographic information form and self-report measures of parenting styles, maladaptive coping styles, and EAB. Model 4 of Hayes’ PROCESS macro in SPSS was employed to identify and explain the mechanism or process that underlies an observed relationship between study variables.

**Results:**

The results showed that authoritarian parenting style, overcompensation and avoidance coping styles, and female gender might be related to disturbed EAB. The overall hypothesis that overcompensation and avoidance coping styles mediated the effect of fathers’ and mothers’ authoritarian parenting styles on disturbed EAB was also supported.

**Conclusions:**

Our findings highlighted the necessity of evaluating particular unhealthy parenting styles and maladaptive coping styles as the important possible risk factors in the development and maintenance of higher level of disturbance in EAB among patients with FED. However, more research is needed to explore individual, family, and peer risk factors for disturbed EAB among these patients.

## Introduction

The main feature of feeding and eating disorders (FED) is the persistent disturbance of eating or eating-related behaviors, which leads to the altered consumption or absorption of food and significantly disturbs physical health or psychosocial functioning (Dalle Grave, 2011). Although the lifetime prevalence of this group of disorders has been reported to be relatively low (0.8% to 11%) (Dahlgren, Wisting & Rø, 2017), factors such as high levels of complexity, heterogeneity, chronicity, ego-syntonicity, and comorbidity with other psychiatric disorders have caused FED to be recognized as one of the most complicated psychopathologies (Brown et al., 2016). Since patients with FED display significantly more disturbed eating attitudes and behaviors (EAB) than healthy subjects, research is required to identify risk factors associated with these attitudes and behaviors to understand the clinical course of FED and also improve physical health or psychosocial functioning among this clinical group (McClelland et al., 2020; Hampshire, Mahoney & Davis, 2022). While empirical findings have supported some of the psychopathological risk factors as potential etiological factors for abnormal EAB, their specific mechanisms remain unknown (McClelland et al., 2020; Hampshire, Mahoney & Davis, 2022; Sheffield et al., 2009).

Nevertheless, in recent decades, numerous theoretical models have been proposed for understanding the development of eating problems among patients with FED. Among them, the most effective model in psychological therapy originates from the cognitive-behavioral tradition (Tetley et al., 2014; Beck et al., 1979). It is presumed in this theoretical framework that dysfunctional beliefs triggered by early negative life experiences cause psychological distress (Tetley et al., 2014; Fairburn, 2008). Despite introducing Cognitive Behavioral Therapy (CBT) as the selective treatment for FED, a significant number of patients do not respond adequately to this therapeutic approach (Brown et al., 2016). As per restricted clinical efficacy of maintenance models, such as CBT in treating FED, particularly for those with higher comorbidity and severity, more additional risk factors are currently being considered by the researchers to improve therapeutic models and the conceptualization and treatment of FED (Cooper & Kelland, 2015; Talbot et al., 2015). In light of these shortcomings, there has recently been a growing interest in examining the utility of schema therapy on eating pathology (Pugh, 2015).

In this respect, a recent FED model suggests that early experiences, such as unhealthy parenting styles, can form negative, unconditional core beliefs (*e.g*., “I am unlovable”) (Sheffield et al., 2009). Such schema-level negative core beliefs might result in the formation of maladaptive coping styles by provoking distressing emotions (Sheffield et al., 2009). Two different types of maladaptive coping styles were introduced by Waller, Kennerley & Ohanian (2007): (i) avoidance coping style (which avoids understanding of intolerable emotional states and cognitions by developing impulsive behaviors, especially binge eating); (ii) overcompensation coping style (which prohibits emotional activation by developing long-term behavioral patterns such as restriction and compulsive exercise). Although these maladaptive coping styles are accompanied by emotional resilience in the short term, they lead to maintaining and exacerbating irrational beliefs and increasing detrimental effects for two main reasons: (i) maladaptive coping styles can prevent someone from challenging their irrational beliefs (*e.g*., “life would not be worth living without bingeing”); (ii) behavioral manifestations of these maladaptive coping styles might be perceived as direct evidence for irrational beliefs (*e.g*., “persistent bingeing can be a sign of my imperfection”) (Sheffield et al., 2009; Waller, Kennerley & Ohanian, 2007). Moreover, since it is possible for children to partially shape their parents’ parenting styles in their behaviors, it will be important to consider this as a possible risk factor for abnormal EAB (Sheffield et al., 2009). In this regard, a systematic review of recent research has demonstrated that parenting styles (*e.g*., authoritative, authoritarian, and permissive parenting styles) have been weakly to moderately associated with individual domains of child feeding, wherein permissive parenting style was negatively associated with monitoring for both fathers and mothers (as the most consistent relationship) (Collins, Duncanson & Burrows, 2014). According to these phenomenological descriptions, the FED model suggests that disturbed EAB can be considered one of the most important manifestations of unhealthy parenting and maladaptive coping styles—a hypothesis supported by recent evidence in several studies (Waller, Kennerley & Ohanian, 2007; Haycraft & Blissett, 2010; Enten & Golan, 2009; Zubatsky, Berge & Neumark-Sztainer, 2015; Peleg, Tzischinsky & Spivak-Lavi, 2021; Collins, Duncanson & Burrows, 2014). In detail, for detached protector/self-soother modes, compensatory and restrictive eating behaviors can act as a form of primary or secondary emotional avoidance while producing soothing feelings of numbness, or euphoria in some cases (Brown et al., 2016). It may identify important mechanisms to target in treating disturbed EAB among patients with FED.

Despite the fact that the model presented above implies a relationship between core beliefs and perceived parental behaviors among patients with FED, very few pieces of comparable evidence have been offered regarding the correlation between parenting styles, maladaptive coping styles, and disturbed EAB in this group of patients (Brown et al., 2016; Sheffield et al., 2009). Accordingly, the present study examines three main psychopathological hypotheses (H) in patients with FED to extend the literature: (H1) parenting styles are associated with disturbed EAB: (H2) avoidance and overcompensation coping styles are related to disturbed EAB; (H3) avoidance and overcompensation coping styles mediated the relationship between parenting styles and disturbed EAB.

## Materials and Methods

### Study design

This cross-sectional study was conducted from April to March 2022 in Zahedan, Iran.

### Sample size calculation

The sample size was estimated at 101 individuals using G*Power software version 3.1.9.4 for 80% power, α error probability of 0.05, medium effect size of 0.20, and 13 predictor variables (Faul et al., 2009; Sheffield et al., 2009), which was increased to 111 participants according to an attrition rate of 10%.

### Participants

Participants were selected through systematic random sampling (with a sampling interval of 3) from among the outpatients referring to Baharan Psychiatric Hospital, Zahedan, Iran. The inclusion criteria were as follows: (i) patients with an above-20 score in the 26-item Eating Attitude Test (EAT-26) (Garner et al., 1982; Ahmadi et al., 2014) whose FED diagnosis was confirmed by an experienced psychiatrist using Structured Clinical Interview for DSM-5 Disorders-Clinician Version (SCID-5-CV) (First et al., 2016); (ii) 18–45 years of age; (iii) minimum literacy and reading comprehension. The exclusion criteria included: (i) severe and acute physical illness; (ii) epileptic disorders; (iii) brain traumatic injury; (iv) intellectual disability; (v) hearing loss; (vi) comorbidity of schizophrenia spectrum and other psychotic disorders; (vii) any substance/medication consumption causing decreased or increased food intake; (viii) incomplete questionnaires.

Finally, a total of 102 patients with FED filled the questionnaires properly (M_age_ = 26.61, SD_age_ = 5.51, Males = 22 (21.6%), Females = 80 (78.4%)) (see Table 1). Besides, a sampling error of 1.5% was also obtained, which suggests that sufficient numbers of samples were taken (Gerbing & Anderson, 1985).

**Table 1:** Socio-demographic factors among study participants (*N* = 102).

Socio-demographic factors	M ± SD	*N* (%)
Age	26.61 ± 5.51	
Gender		
Male		22 (21.6)
Female		80 (78.4)
Marital status		
Single		71 (69.6)
Married		31 (30.4)
Education level		
Non-degree		40 (39.2)
High school diploma		39 (38.2)
Academic degree		23 (22.6)
Income		
<20,000,000 Rials		70 (68.6)
≥20,000,000 Rials		32 (31.4)
BMI		
Underweight		11 (10.8)
Normal range		66 (64.7)
Overweight		12 (11.8)
Obese (Class I)		5 (4.9)
Obese (Class II)		5 (4.9)
Obese (Class III)		3 (2.9)
Feeding and eating disorders		
Anorexia nervosa		2 (2.0)
Bulimia nervosa		4 (3.9)
Binge-eating disorder		7 (6.9)
Other specified feeding or eating disorders		79 (77.4)
Avoidant/restricted food intake disorder		7 (6.9)
Rumination disorder		3 (2.9)

**Notes:**

BMI, body mass index.

BMI classification (kg/m^2^): underweight, <18.5; normal range, 18.5–24.9; overweight, 25.0–29.9; obese (Class I), 30.0–34.9; obese (Class II), 35.0–39.9; obese (Class III), ≥40.0.

### Procedure

After obtaining the Research Ethics Certificate from the ethics committee of the Medical Faculty of the Zahedan University of Medical Sciences (Approval ID: IR.ZAUMS.REC.1399.521), informed consent forms and necessary information about the study’s objectives were given to the participants. After having obtained informed consent forms from participants, an experienced psychiatrist evaluated all of the participants using EAT-26. The sociodemographic information form (including age, gender, marital status, education level, and income), Young Compensation Inventory (YCI) (Young, 1995; Noorbala, Bahram Ehsan & Alipour, 2016), Young-Rygh Avoidance Inventory (Y-RAI) (Young, 1994; Sa’adati et al., 2017), and Buri’s Parental Authority Questionnaire (BPAQ) (Buri, 1991; Besharat, Azizi & Poursharifi, 2011) were only given to the patients with an above-20 score in the EAT-26 (Garner et al., 1982; Ahmadi et al., 2014) whose FED diagnosis was confirmed using SCID-5-CV (First et al., 2016). Further, to abide by the Helsinki declaration (Goodyear, Krleza-Jeric & Lemmens, 2007), the individuals were told that their participation was voluntary and they could leave the study for any given reason. Also, all participants were assured of the ethical principles of confidentiality.

### Instruments

The Persian versions of the following instruments were used in this study (in general, the Cronbach’s alpha values of 0.70 or higher indicate acceptable internal consistency) (Taber, 2018):

#### EAT-26

The EAT-26 is a 26-item questionnaire that includes three subscales of dieting (*i.e*., “scrutiny of calorie content, carbohydrates, and sugar content, motivated by a desire to be thinner”) bulimia and food preoccupation (*i.e*., “the tendency to purge after meals as well as excessive food-related thinking”), and oral control (*i.e*., “the tendency toward self-control of eating”) (Papini et al., 2022). The entire range of EAT-26 scores is from 0 to 78, wherein scores of $\ge$20 denote the probability of having FED (Garner et al., 1982). The version used in the study was a translated version from the one in Ahmadi et al. (2014) article. The reliability and validity of this version of EAT-26 were reported to be suitable (Ahmadi et al., 2014). In our study, the Cronbach’s alpha coefficient for the EAT-26 total scale was 0.90.

#### SCID-5-CV

The SCID-5-CV is a structured interview for major Diagnostic and Statistical Manual of Mental Disorders, Fifth Edition (DSM-5) diagnoses. It is conducted by a trained clinician or health expert acquainted with the classification of disorders and diagnostic criteria in DSM-5. The reliability and validity of SCID-5-CV have been reported acceptable by several studies (First et al., 2016).

#### YCI

This 48-item inventory aims to evaluate overcompensation coping style, which is scored on a six-point (1–6) Likert scale. The minimum and maximum scores in this inventory are 48 and 288, respectively (Young, 1995). The version used in the study was a translated version from the one in Noorbala, Bahram Ehsan & Alipour (2016) article. The reliability and validity of this inventory have been identified as suitable (Cronbach’s alpha = 0.82) (Noorbala, Bahram Ehsan & Alipour, 2016). In the present study, the Cronbach’s alpha coefficient for the YCI total scale was reported to be 0.85.

#### Y-RAI

This inventory includes 40 items aiming to investigate avoidance coping style, which is scored on a five-point (1–5) Likert scale. The minimum and maximum scores in this questionnaire are 40 and 200, respectively (Young, 1994). The version used in the study was a translated version from the one in the study by Sa’adati et al. (2017). The authors reported the reliability and validity of this inventory to be suitable (the Cronbach’s alpha coefficient was 0.79). In the present study, the Cronbach’s alpha coefficient for the Y-RAI total scale was reported 0.82.

#### BPAQ

This questionnaire is composed of 30 items and three subscales of permissive parenting style, authoritarian parenting style, and authoritative/flexible parenting style. Also, father and mother forms are the same, except for appropriate references to the words “father” or “mother”. This questionnaire is scored on a five-point (1–5) Likert scale. An individual’s minimum and maximum scores in each subscale are 10 and 50, respectively (Buri, 1991). The version used in the study was a translated version from the one in the study by Besharat, Azizi & Poursharifi (2011), wherein participants answered the questions retrospectively. The reliability and validity of this questionnaire were reported to be suitable (Besharat, Azizi & Poursharifi, 2011). In our study, the Cronbach’s alpha coefficients were 0.72, 0.85, and 0.79 for permissive parenting style, authoritarian parenting style, and authoritative/flexible parenting style, respectively.

### Statistical analysis

Initially, the normality of data was evaluated by SPSS v25.0 software, and the mean scores of each variable were investigated by the descriptive statistical analysis. According to the acceptable range for normality in the formal normality tests (including the Shapiro-Wilk test and Kolmogorov-Smirnov test), point-biserial correlation coefficient, Pearson’s r, Spearman’s ρ, Gamma, Cramer’s V, and multiple regression analysis (enter method) were conducted to investigate the relationship between explanatory variables (*i.e*., the mediating roles of overcompensation and avoidance coping styles and parenting styles) and response variable (*i.e*., disturbed EAB) (H1 and H2). Mediation analysis with bootstrap sampling was performed to assess the mediating role of overcompensation and avoidance coping styles in the relationship between parenting styles and disturbed EAB (by controlling the effect of clinical features and sociodemographic factors) (H3). As suggested by Preacher & Hayes (2004), the bootstrap method (with 5,000 bootstrap samples and 95% bias-corrected confidence intervals) was used to estimate direct, indirect, and total effects. Ordinary least squares regression was used to estimate all paths. These analyses were performed by the Hayes’ PROCESS macro method v3.4.1, a computational procedure for SPSS (Hayes, 2017). As stated by Preacher & Hayes (2004), there is a mediating role provided that the confidence interval (CI) does not include a zero value and the indirect effect is significant. In addition, only variables with statistically significant correlation with disturbed EAB among patients with FED were included in the mediation analysis.

## Results

### H1 and H2: factors associated with disturbed EAB

The results obtained from the correlation matrix revealed that disturbed EAB had a positive and significant correlation with gender (r = 0.21, *p* = 0.031) and scores of father’s authoritarian parenting style (r = 0.53, *p* < 0.001), mother’s authoritarian parenting style (r = 0.59, *p* < 0.001), overcompensation coping style (r = 0.76, *p* < 0.001), and avoidance coping style (r = 0.77, *p* < 0.001) (see Table 2).

**Table 2:** A correlation matrix between overall Eating Attitude Test-26 scores and potential independent variables among patients with feeding and eating disorders (*N* = 102).

Variables	1	2	3	4	5	6	7	8	9		10	11	12	13	14	15
1. Age	–															
2. Gender	−0.05	–														
3. MS	0.41***	−0.17	–													
4. EL	0.12	0.34	0.12	–												
5. Income	0.53***	−0.37	0.49*	0.02	–											
6. BMI	−0.17	−0.13	−0.03	−0.05	0.05	–										
7. PPS (F)	0.09	0.14	−0.04	−0.04	−0.05	−0.18	–									
8. PPS (M)	0.07	0.01	0.04	0.08	−0.07	−0.11	0.19*	–								
9. APS (F)	−0.11	0.10	−0.15	−0.00	−0.04	−0.06	−0.20*	0.08	–							
10. APS (M)	−0.01	0.09	0.03	0.03	−0.01	−0.00	0.06	−0.15	0.36***		–					
11. A/FPS (F)	−0.22*	0.04	−0.05	0.10	−0.22*	0.15	0.04	0.01	−0.13		0.03	–				
12. A/FPS (M)	−0.16	0.19	−0.10	0.17	−0.24*	−0.26**	0.14	0.21*	0.06		−0.13	0.14	–			
13. OCS	−0.16	0.11	−0.12	0.07	−0.14	0.00	−0.04	−0.09	0.49***		0.46***	−0.06	−0.00	–		
14. ACS	−0.14	0.07	−0.10	0.08	−0.04	0.01	−0.03	−0.08	0.50***		0.51***	−0.03	−0.01	0.83***	–	
15. EAT-26	−0.14	0.21*	−0.11	0.06	−0.10	0.09	0.05	−0.02	0.53***	0.59***		0.03	0.03	0.76***	0.77***	–

**Notes:**

Point-biserial correlation coefficient, Pearson’s r, Spearman’s ρ, Gamma, and Cramer’s V were used to examine the correlations between study variables.

ACS, avoidance coping style; A/FPS, authoritative/flexible parenting style; APS, authoritarian parenting style; BMI, body mass index; EAT-26, the 26-item Eating Attitude Test; EL, education level; F, father; M, mother; MS, marital status; OCS, overcompensation coping style; PPS, permissive parenting style.

* *p* < 0.05.

** *p* < 0.01.

*** *p* < 0.001.

Table 3 summarizes the results of the multiple regression analysis with the enter method. As can be seen, gender (β = 0.12, *p* = 0.033), father’s authoritarian parenting style (β = 0.13, *p* = 0.048), mother’s authoritarian parenting style (β = 0.22, *p* = 0.001), overcompensation coping style (β = 0.31, *p* = 0.011), and avoidance coping style (β = 0.31, *p* = 0.012) could account for 70% of the total variance in EAT-26 scores among study participants (F(5, 96) = 46.81, *p* < 0.001). These findings indicate that the female gender and high scores on father’s authoritarian parenting style, mother’s authoritarian parenting style, overcompensation coping style, and avoidance coping style are likely to be associated with disturbed EAB.

**Table 3:** Multiple linear regression analysis predicting total scores of EAT-26 as a function of gender, APS (F), APS (M), OCS, and ACS in patients with feeding and eating disorders (*N* = 102).

Response variable	Explanatory variables	B	SE	β	95% CI
EAT-26	Gender	3.55	1.64	0.12*	[0.29–6.81]
	APS (F)	0.14	0.07	0.13*	[0.00–0.28]
	APS (M)	0.24	0.07	0.22**	[0.10–0.38]
	OCS	0.07	0.02	0.31*	[0.01–0.13]
	ACS	0.08	0.03	0.31*	[0.01–0.15]
R^2^ = 0.70, Adjusted R^2^ = 0.69, F (5, 96) = 46.81***					

**Notes:**

ACS, avoidance coping style; APS, authoritarian parenting style; CI, confidence interval; EAT-26, the 26-item Eating Attitude Test; F, father; M, mother; OCS, overcompensation coping style; SE, standard error.

* *p* < 0.05.

** *p* < 0.01.

*** *p* < 0.001.

### H3: mediation analysis

**Figure 1:**
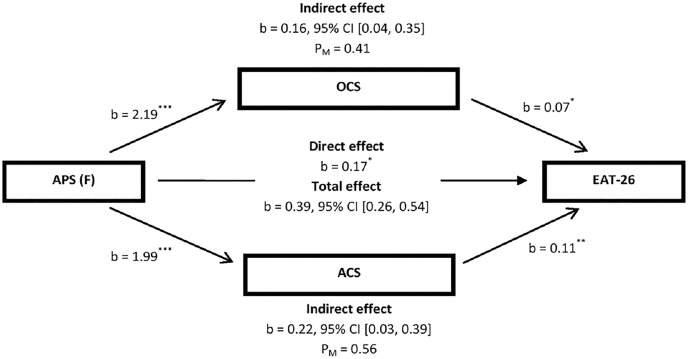
Illustration of the results of the mediation analysis described in the text, which tested OCS (*i.e*., overall YCI scores) and ACS (*i.e*., overall Y-RAI scores) as the potential mediators of the relationship between APS (F) and overall EAT-26 scores by controlling for gender among patients with feeding and eating disorders (*N* = 102). ACS, avoidance coping style; APS, authoritarian parenting style; CI, confidence interval; EAT-26, the 26-item Eating Attitude Test; F, father; OCS, overcompensation coping style; YCI, young compensation inventory; Y-RAI, Young-Rygh avoidance inventory. P_M_, effect size (ratio of indirect to total effect). *p < 0.05; **p < 0.01; ***p < 0.001.

In the mediation model presented in Fig. 1, a significant indirect effect of father’s authoritarian parenting style was distinctly observed on disturbed EAB through overcompensation coping style (*i.e*., overall YCI scores) and avoidance coping style (*i.e*., overall Y-RAI scores) (b = 0.16, 95% CI [0.04–0.35]; b = 0.22, 95% CI [0.03–0.39], respectively). The mediators, *i.e*., overcompensation coping style and avoidance coping style, could account for 41% and 56%, respectively, of the total effect of the father’s authoritarian parenting style on disturbed EAB. Hence, the overall hypothesis that overcompensation coping style and avoidance coping style partially mediate the effect of the father’s authoritarian parenting style on disturbed EAB was supported.

**Figure 2:**
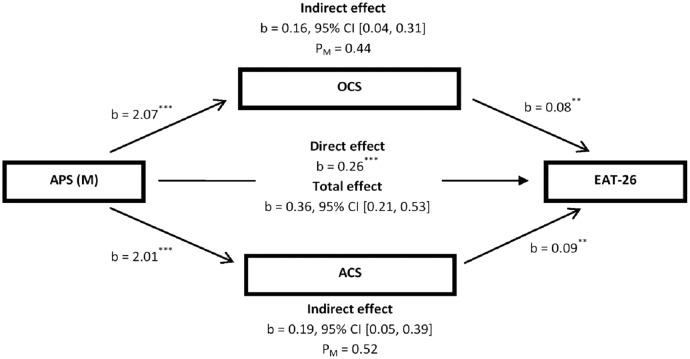
Illustration of the results of the mediation analysis described in the text, which tested OCS (*i.e*., overall YCI scores) and ACS (*i.e*., overall Y-RAI scores) as the potential mediators of the relationship between APS (M) and overall EAT-26 scores by controlling for gender among patients with feeding and eating disorders (*N* = 102). ACS, avoidance coping style; APS, authoritarian parenting style; CI, confidence interval; EAT-26, the 26-item Eating Attitude Test; M, mother; OCS, overcompensation coping style; YCI, young compensation inventory; Y-RAI, Young-Rygh avoidance inventory. P_M_, effect size (ratio of indirect to total effect). **p < 0.01; ***p < 0.001.

In the mediation model presented in Fig. 2, a significant indirect effect of mother’s authoritarian parenting style was distinctly noticed on disturbed EAB through overcompensation coping style (*i.e*., overall YCI scores) and avoidance coping style (*i.e*., overall Y-RAI scores) (b = 0.16, 95% CI [0.04–0.31]; b = 0.19, 95% CI [0.05–0.39], respectively). In this mediation model, overcompensation coping style and avoidance coping style also could account for 44% and 52%, respectively, of the total effect of the mother’s authoritarian parenting style on disturbed EAB. Accordingly, the overall hypothesis that overcompensation coping style and avoidance coping style partially mediated the effect of mother’s authoritarian parenting style on disturbed EAB was also supported.

## Discussion

The present research is among the few studies conducted for an appropriate sample size of outpatients with FED to examine a part of a new schema-focused cognitive-behavioral model for eating psychopathology—a model suggesting that maladaptive coping styles such as overcompensation coping style and avoidance coping style might mediate the relationship between parenting styles and disturbed EAB (Waller, Kennerley & Ohanian, 2007).

When addressing H1, our results illustrated that the fathers’ and mothers’ authoritarian parenting styles were positively related to disturbed EAB. In other words, children’s engagement with severe weight control behaviors is created by parents exhibiting more demanding (*e.g*., limits/expectation) and less responsive (*e.g*., connectedness/communication) parenting styles (Zubatsky, Berge & Neumark-Sztainer, 2015). However, in our study, disturbed EAB were not significantly associated with permissive parenting style and authoritative/flexible parenting style. These results imply the presence of mixed findings regarding the effects of fathers’ and mothers’ parenting styles on offsprings’ eating behaviors and patterns. For instance, some previous studies suggested that a father’s or mother’s authoritative style might play a protective role in the formation of a child’s disturbed EAB and FED (Olvera & Power, 2010; Rhee et al., 2006; Berge et al., 2010), whereas the preliminary findings of a recent longitudinal study have not shown such a relationship between fathers’ parenting style and offsprings’ disturbed EAB (Zubatsky, Berge & Neumark-Sztainer, 2015). The inconsistency of these results might be due to the difference in conceptualization and measurement of parenting styles. Vollmer & Mobley (2013) showed in their review that the studies on parenting style made use of 10 various validated measures; however, other studies employed invalidated measures introduced by specific authors, resulting in varied conceptualizations of parenting style and dimensions. Moreover, they conceptualized the responsiveness dimension differently. These changes in conceptualization and measurements of style dimensions have significantly hampered the possibility to realize the relationship between disturbed EAB and parenting style.

When addressing H2, our results revealed that overcompensation coping style and avoidance coping style were positively related to disturbed EAB. These results are in general agreement with previous data, showing that disturbed EAB might function as primary avoidance strategies (such as (i) restricting, purging, or over-exercising in a compulsive or ritualized manner; (ii) reducing the chance of schema activation) or secondary avoidance strategies (*e.g*., minimizing the emotional distress caused by bingeing or other dietary transgressions) (Waller, Kennerley & Ohanian, 2007; Brewerton et al., 1995; Simpson, 2016; Simpson, 2012; Davis et al., 1997; Thien et al., 2000; Hechler et al., 2005). Further, these findings support the previous research that has confirmed the overcompensatory role of disturbed EAB (Boone et al., 2013; Mountford et al., 2004). To explain the results, a schema-based cognitive–behavioral model of FED pathology has shown that the process of schema compensation has a key role in the development of eating pathologies. In detail, when there is a risk of experiencing negative affect, compensatory schemas are activated to reduce affect (Talbot et al., 2015; Voderholzer et al., 2014).

As another interesting finding of this study, the results of the multiple regression analysis indicated a significant association between female gender and higher levels of disturbance in EAB among patients with FED. Since analyses concerning gendered parenting styles and development of disturbed EAB were not conducted, future research should address the possible relationship between gendered parenting styles and disturbed EAB among patients with FED. However, the gender-related difference in the level of disturbance in EAB may be explained by social factors (*e.g*., different expectations about cultural beauty standards) and biological factors (*e.g*., levels of ovarian hormones) (Culbert, Racine & Klump, 2015; Zubatsky, Berge & Neumark-Sztainer, 2015; Rolls, Fedoroff & Guthrie, 1991).

Moreover, the results of our study showed no significant relationship between disturbed EAB and age. These findings are inconsistent with the results obtained by a meta-analysis on the changes in disturbed EAB in the long term that implies the risk of engaging in various restrictive eating and other compensatory behaviors decreases over time (Romano et al., 2020). This contradiction might be attributed to our large number of participants in the young adult age category. Besides, analogous to Fitzsimmons-Craft et al. (2019) and Lantz et al. (2017), we found no significant correlation between disturbed EAB and BMI (as the other covariate). These findings demonstrate that BMI alone might be an insufficient marker of future disturbed EAB risk at specific time periods (Romano et al., 2020).

When addressing H3, our results indicated a significant indirect effect of fathers’ and mothers’ authoritarian parenting styles on disturbed EAB through overcompensation coping style and avoidance coping style. These data are compatible with the model in which behavioral-somatic avoidance is a partial mediator in the relationship between emotionally inhibited mothers and body dissatisfaction (Sheffield et al., 2009). A potential explanation for this link is that behavioral-somatic avoidance is developmentally a more prominent and dysfunctional schema process, which is measured by Y-RAI (Spranger, Waller & Bryant-Waugh, 2001). Although the cognitive capacity and the ability to employ more complicated cognitive/emotional strategies for controlling emotions are restricted in children, they probably learn from an early age that withdrawing from a situation or being ill helps them evade from unpleasant emotions (Sheffield et al., 2009). Basically, authoritarian parents are not physically affectionate and avoid sharing their emotions or vulnerabilities. Therefore, the authoritarian parenting style does not seem necessary to serve as an effective pattern to develop coping mechanisms for concerns or validate the child’s emotions. As a result, the child does not manage to learn constructive approaches to deal with unpleasant emotions and is left later in life to take advantage of behavioral-somatic avoidance (Spranger, Waller & Bryant-Waugh, 2001; Sheffield et al., 2009). When more robust approaches to experience and cope with difficult emotions are not available, the odds are that the individual is more fearful of life experiences and the subsequently evoked emotions (Sheffield et al., 2009; Brown et al., 2016). Thus, they do not experience, tolerate and cope more adaptively with emotions, but rather they might transfer their anxieties or fears into something more tangible and possibly controllable (*e.g*., dissatisfaction with the body) (Sheffield et al., 2009). Further, according to the restrictive behavior model, one can propose the hypothesis that the activation of negative schema-level beliefs among patients with FED initiates compensatory cognitions and a range of different compensatory behaviors, which can lead to the activation of a number of potential compensatory schemas (*e.g*., perfectionist behavior) depending on developmental experience and the nature of the triggering core belief (Mountford et al., 2004; Shafran & Mansell, 2001).

### Limitations and future directions

In the current study, some factors limit the robustness and generalizability of the presented findings. As the first limitation, the study data were the responses of 102 young adult participants, which cannot be generalized to various cases. Thus, a large, diverse, population-based sample might increase the power of the link between variables. As the second restriction, key instruments used in this study were self-report questionnaires, which are not able to show the real emotions of participants. Additionally, there might have been effects of retrospective reporting on parenting. For example, individuals with high levels of eating pathology are biased in their reports of parenting and are more likely to have negative views of their childhood, which makes it difficult to accurately represent the parenting perceived as a child. Accordingly, as a sensible suggestion, self-report measures should be used alongside other reporting methods in future studies since they suffer from a lack of empirical data, memory bias and demand characteristics, and disregarding ethnic differences. As the third limitation, it is not feasible to make proper assumptions about causality based on cross-sectional data, including recalled childhood experiences. Complex interactions exist between eating pathology, perceived parenting behavior, and actual parenting behavior. For instance, individuals’ recall of their perceptions in our study might be affected by their more recent experience with their parents, recall bias, the processes they use for defending against painful experiences (*e.g*., idealization or avoidance), and filters currently in operation (*e.g*., core beliefs). Accordingly, a longitudinal design would offer more insight into the roles of early parenting experiences and maladaptive coping styles as the important possible risk factors in the development and maintenance of higher levels of disturbance in EAB among patients with FED. As the fourth restriction, the participants were not asked about other personal characteristics since, based on the available evidence (Sheffield et al., 2009; Enten & Golan, 2009), many mediating and moderating factors like temperament, self-esteem, and depression might be involved in the perceptions of parental behavior and development of eating pathology. Therefore, future studies can be enhanced by considering the hypotheses about the distinct roles of these causal factors. As the fifth limitation, this study did not investigate disturbed EAB in participants’ parents. If fathers or mothers have a history of weight control issues or FED, the onset of weight control behaviors of offsprings will be affected (Lenfant et al., 2009). Finally, most of our sample had a diagnosis of “other specified feeding or eating disorders”, a highly heterogeneous diagnosis. Hence, the heterogeneity of this sample might limit the conclusions we drew. Future studies might need to focus on particular FED subtypes.

### Clinical implications

Despite the above limitations, our study obtained a preliminary comprehension of the effects of parenting styles and schema processes on the level of disturbance in EAB among patients with FED. The clinical implications of the present study can be divided into two major parts. Firstly, our results revealed that the identification of the authoritarian parenting style might be perceived as an important target for early interventions to prevent disturbed EAB and clinical course of FED in offspring. Besides, these results suggest that treatment programs should address schema processes as one of the critical, possible factors in the development and maintenance of disturbed EAB. This approach could include behavioral strategies (such as encouraging direct experimentation with more adaptive techniques to cope with unpleasant emotions), cognitive strategies (namely, examining the short- and long-term effects of avoidant, compensatory, and blocking behaviors), and experimental strategies. The links among schema processes, perceived parenting, and disturbed EAB should be better understood to help clients challenge how they manage unpleasant emotions. Accordingly, self-blame can be reduced, which allows patients with FED to realize that their behavior can be understood and was likely to be adaptively functional in earlier environments (Kennerley, 1996; Young, Klosko & Weishaar, 2006).

## Conclusions

This study was one of only a few to collectively examine the relationship between parenting styles, maladaptive coping styles, and disturbed EAB among patients with FED. Based on our findings, authoritarian parenting style and maladaptive coping styles (*e.g*., avoidance and overcompensation) seem to play a critical role in developing and maintaining of disturbed EAB among patients with FED. This highlights the significance of the potential role of early interventions and early experiences for patients with FED and their families as key to avoiding the development of dysfunctional schema modes that may result in the formation of disturbed EAB. The very large number of significant pathways between schema modes, particular parenting styles, and bingeing, restricting, and compensatory behavior also suggest the complexity of disturbed EAB psychopathology. This highlights the necessity to adopt more individualized approaches (instead of just one-size-fits-all treatment approaches) with the capability to identify and work with the (historical) functional facet of disturbed EAB closely related to schema modes, while underlining their self-sabotaging role in the present. Schema Therapy not only helps more adaptive and effective coping behaviors with a direct focus on fulfilling emotional needs to be developed but also, reduces reliance on disturbed EAB coping behaviors. Future research is required to replicate the current findings and investigate the efficacy of schema-mode therapy in treating disturbed EAB.

## Supplemental Information

10.7717/peerj.14880/supp-1 Supplemental Information 1 Data for analysis.

## Additional Information and Declarations

### Data Availability

The following information was supplied regarding data availability: The raw measurements are available as a Supplemental File.
